# Menopause and “leaky gut”: implications for midlife women’s health

**DOI:** 10.1172/JCI209807

**Published:** 2026-09-01

**Authors:** Brandilyn A. Peters

**Affiliations:** Department of Epidemiology and Population Health, Albert Einstein College of Medicine, Bronx, New York, New York, USA.

## Abstract

Menopause may have important consequences for gut barrier health. The roles of estradiol and progesterone in immune and mucosal barrier homeostasis have been well studied in the female reproductive tract. However, few human studies have described these hormones’ corresponding regulation in the gastrointestinal tract or how the menopausal transition affects gut barrier integrity. In this issue of the *JCI*, Shieh et al. report that markers of gut epithelial barrier dysfunction and microbial translocation–related immune activation increased across the menopause transition in a longitudinal study of healthy women. These findings suggest that ovarian aging may contribute to gut barrier dysfunction in midlife women and highlight new questions about clinical consequences and potential interventions.

## What is “leaky gut”?

In the healthy gastrointestinal tract, several layers of defense preserve the integrity of the gut barrier, including mucus, epithelial barriers, and resident macrophages (1, 2). However, when gut barrier integrity is compromised — a phenomenon often referred to as “leaky gut” — microbial products may translocate from the intestinal lumen into the circulation (i.e., microbial translocation), leading to deleterious immune activation and inflammation (1, 2). Gut barrier dysfunction has been described across a range of physiologic, stress-related, and disease states, including aging, pregnancy, endurance exercise, inflammatory bowel disease, and liver disease (2).

## Estrogen, progesterone, and gut permeability

A body of experimental work supports a role for the ovarian sex hormones, estradiol and progesterone, in preserving intestinal barrier integrity and mitigating gut injury. In vitro and ex vivo studies have shown that intestines from female rats are less susceptible to shock-induced injury than those from male rats, an effect that can be restored in males with estradiol treatment (3). Estradiol also protects mucus-secreting intestinal epithelial cells from oxidant-mediated damage (4). Additional studies indicate that estrogen receptor-β contributes to maintenance of colonic epithelial barrier function (5, 6), while both estradiol and progesterone enhance epithelial barrier function in intestinal epithelial cell models through increased expression of tight junction proteins (7, 8). Consistent with these findings, ovariectomy increases intestinal permeability in mice (9). Observational human data further suggest relevance of estradiol and progesterone to gut barrier health: estrogen receptor-β mRNA expression is decreased in the colonic tissue of patients with inflammatory bowel disease (5), and during human pregnancy, circulating progesterone concentrations are inversely associated with plasma lipopolysaccharide (LPS), a component of the outer membrane of Gram-negative bacteria and marker of microbial translocation (8, 10). Collectively, these findings support the concept that ovarian hormones, particularly estradiol and progesterone, help limit intestinal permeability and microbial translocation.

Estradiol and progesterone are widely recognized as key regulators of immune function and mucosal barrier homeostasis in the female reproductive tract. Declines in these hormones during menopause contribute to vaginal symptoms and heightened vulnerability to pathogenic infections (11, 12). A similar hormone-dependent mechanism may operate in the gastrointestinal tract, whereby menopause-related reductions in ovarian hormones compromise gut barrier integrity and promote microbial translocation. However, relatively few human studies have directly evaluated the relationship among menopause, intestinal barrier function, and microbial translocation.

## Leaky gut during menopause

In 2020, Shieh et al. published the first human study to examine the association of menopause with gut barrier outcomes (13) within the Study of Women’s Health Across the Nation (SWAN), a multiethnic US cohort designed to examine the health of women during menopause. In that study, plasma intestinal fatty acid binding protein 2 (FABP2), a marker of gut epithelial cell damage; lipopolysaccharide binding protein (LBP), a marker of microbial translocation; and soluble CD14 (sCD14), a marker of microbial translocation–related immune activation, all increased from pre- to post-menopause among 65 women (13). Additionally, an inverse association was observed between plasma estradiol and FABP2 and sCD14 levels. These results supported the possibility that microbial translocation increases during the menopause transition in association with declining sex hormone levels, but the observations could not be fully separated from aging-related changes.

In this issue of the *JCI*, Shieh et al. follow up on their previous findings to conduct a more comprehensive longitudinal study of gut barrier dysfunction across the menopause transition in the SWAN (14). In this new study, serum FABP2 and sCD14 were measured among 964 women at 3,586 time points spanning before, during, and after the menopause transition (median: 4 samples per participant; minimum 2 and maximum 7 samples per participant). The main analysis examined longitudinal changes in FABP2 and sCD14 in time segments defined by time before and after the final menstrual period (FMP). This modeling strategy, employed using piecewise linear mixed effects models, is considered the gold standard to differentiate whether biomarkers increase at a constant rate over time, consistent with chronological aging, or whether they accelerate during the menopause transition, suggesting an effect of ovarian aging beyond that of chronological aging. Their results indicated that FABP2 and sCD14 did indeed increase over time during the menopause transition (~2 years before to ~6 years after the FMP), but did not change over time before or after this period, consistent with an effect of ovarian aging on gut barrier dysfunction. Cumulatively, FABP2 and sCD14 increased by 26% and 7.5% during the menopause transition, respectively, and results did not differ by race/ethnicity, BMI, or age at FMP. Further, mediation analysis suggested that increase in FABP2 partly explained the increase in sCD14 during the menopause transition. Taken together, these findings provide the strongest evidence to date that menopause may promote gut barrier dysfunction and microbial translocation in otherwise-healthy women.

## Strengths and limitations of the current research

**Figure 1:**
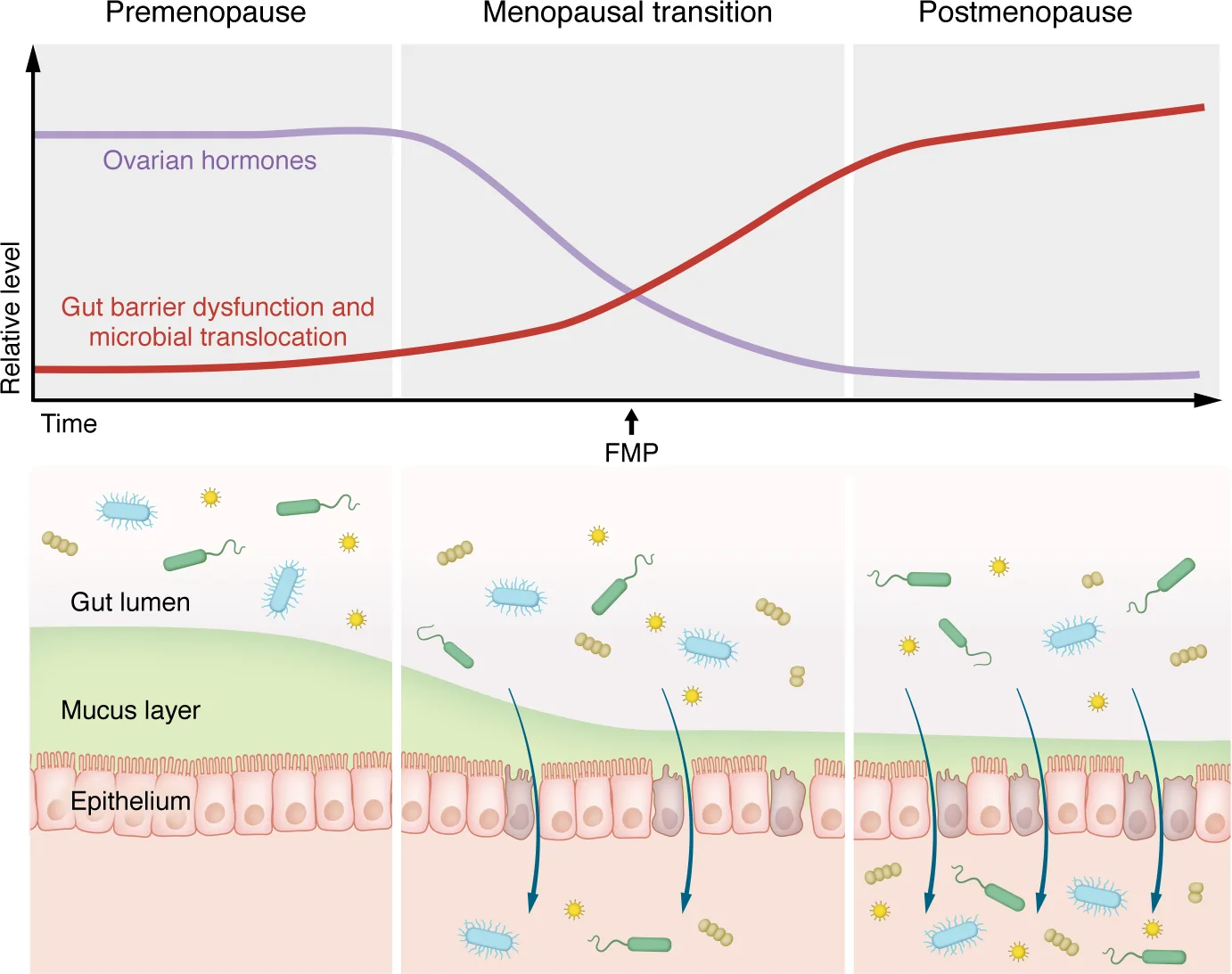
Proposed model linking menopause-related ovarian hormone decline with gut barrier dysfunction and microbial translocation. During the menopause transition, circulating ovarian hormones (estradiol and progesterone) decline. Experimental studies suggest that these hormones support intestinal epithelial barrier integrity, while longitudinal data from Shieh et al. (14) showed increases in markers of gut epithelial barrier compromise and microbial translocation–related immune activation across the menopause transition. An arrow marks the proposed time point when the final menstrual period (FMP) occurs relative to these hormonal and gut barrier changes.

The design of the current study by Shieh et al. (14), featuring a large sample size of generally healthy women with repeated measures of gut barrier–related biomarkers spanning before, during, and after the menopause transition, is ideal for examining the question at hand, of whether gut barrier dysfunction and microbial translocation increase during menopause. Having answered this question, several limitations remain. First, the study was unable to measure a direct biomarker of microbial translocation (e.g., LPS), or the first-response acute-phase protein to LPS translocation (LBP), and makes inference based on FABP2, which reflects intestinal epithelial cell injury or turnover rather than permeability directly, and sCD14, an indirect, nonspecific marker of monocyte activation that can reflect, but is not unique to, microbial translocation. The authors noted that sCD14 was measured in this study in lieu of LBP, due to the stronger inverse correlation of plasma estradiol with sCD14 in their pilot study (13), while LPS was not measured due to concerns of sample bacterial contamination, which could inflate LPS levels (14). Second, the analysis did not address the association of hormone levels (e.g., estradiol, progesterone) with the gut barrier and microbial translocation biomarkers, which would provide further understanding of the mechanism of gut barrier dysfunction during menopause. Nevertheless, the intriguing results combined with prior literature indicating the protective role of estradiol and progesterone on the gut barrier support the hypothesis that declines in ovarian hormones during the menopause transition may contribute to gut barrier dysfunction, intestinal permeability, and microbial translocation (Figure 1).

## Questions and clinical implications for midlife women’s health

The observed increase in gut barrier dysfunction and microbial translocation during the menopause transition in generally healthy women raises a number of practical clinical questions and concerns for menopausal women and their health care providers. What are the clinical consequences of a menopause-related increase in microbial translocation? In principle, microbial translocation causes immune activation and inflammation, a precursor for many diseases; could gut barrier dysfunction during menopause contribute to menopause-related disease risks, such as cardiovascular disease and osteoporosis? If so, would therapies to improve gut barrier function, such as probiotics (15), help mitigate menopause-related disease? Could menopausal hormone therapy — typically estrogen with or without progesterone, indicated for treatment of menopausal symptoms such as vasomotor symptoms and prevention of bone loss (16) — also improve gut barrier function if taken during the menopause transition and lessen the related health consequences? Does gut barrier dysfunction during menopause also contribute to mounting reports of elevated gastrointestinal symptoms during menopause (17, 18)? With the topic of menopause rising to prominence in social and lay media (19), coinciding with growing calls for expanded research (20, 21), the time is ripe to begin answering these questions using rigorous longitudinal epidemiology studies and clinical trials. More research on the connection of menopause with gut barrier health can ultimately provide guidance on actionable steps for improving health and preventing disease for women during the critical time of the menopause transition.

## Conflict of interest

The author has declared that no conflict of interest exists.

## Funding support

This work is the result of NIH funding, in whole or in part, and is subject to the NIH Public Access Policy. Through acceptance of this federal funding, the NIH has been given a right to make the work publicly available in PubMed Central.

- BAP by the NIH NHLBI (R03HL182350).

## Version 1 09/01/2026

Electronic publication
